# Resource efficiency and environmental sustainability of wheat production in Türkiye

**DOI:** 10.1038/s41598-025-31498-w

**Published:** 2025-12-05

**Authors:** Müjdat Öztürk, Hasan Yildizhan, Arman Ameen

**Affiliations:** 1 Department of Mechanical Engineering, Faculty of Engineering and Architecture, Kırşehir Ahi Evran University, Kırşehir, Türkiye; 2 Department of Energy Systems Engineering, Adana Alparslan Türkeş Science and Technology University, Adana, Türkiye; 3 Department of Building Engineering, Energy Systems and Sustainability Science, University of Gävle, Gävle, 801 76 Sweden

**Keywords:** Wheat production, Renewability indicator, Sustainable agriculture, Energy and exergy utilization, Türkiye, Climate sciences, Ecology, Ecology, Environmental sciences, Environmental social sciences

## Abstract

The environmental impact of agricultural production varies depending on input levels. This study provides a comparative sustainability assessment of wheat production in two different provinces of Türkiye, Samsun and Tokat, by examining the energy, exergy and environmental performance on a per ton basis. Based on exergy consumption, Cumulative Degree of Perfection (CDP) and Renewability Index (RI) indicators were determined. The results show that Cumulative Energy Consumption (CEnC) is 7262.93 MJ/ton in Samsun and 3502.97 MJ/ton in Tokat. This indicates that wheat production in Samsun is approximately twice as energy intensive as in Tokat. Cumulative Exergy Consumption (CExC) was calculated as 10514.76 MJ/ton in Samsun and 5400.88 MJ/ton in Tokat. Here, the largest component of the exergy load is irrigation, followed by diesel consumption. From an environmental perspective, Cumulative CO₂ Emissions (CCO_2_E) was found to be 957.5 kg/ton in Samsun and 562.27 kg/ton in Tokat. The sustainability metrics, CDP and RI values, were calculated as 2.13 and 0.53 for Samsun and 4.14 and 0.76 for Tokat, respectively. Based on these findings, it is evident that Samsun has lower exergetic efficiency and a limited degree of renewability due to higher fuel and irrigation inputs. These results suggest that Tokat presents a more sustainable model for wheat production.

## Introduction

Over the last century, the rapid growth of the global population has led to a significant increase in food demand and energy use. This has pushed the agricultural sector to produce more food by relying heavily on fossil fuels. Traditional farming methods are highly dependent on these energy intensive fossil fuels to meet rising demand. The limited and diminishing reserves of fossil fuels make it essential to adopt sustainable methods in agricultural production. In this context, improving energy efficiency in agriculture is crucial for both economic sustainability and environmental protection.

Research on agricultural production processes shows that a large portion of the energy consumed comes from non-renewable sources, particularly diesel fuel and chemical fertilizers^1,2^. The excessive use of these inputs not only reduces the sustainability of the system but also contributes to greenhouse gas emissions, leading to biodiversity loss and water and soil pollution. Therefore, input and output analyses are widely used to assess energy use efficiency, environmental impact and sustainability in agricultural systems^3–5^.

As a staple food for the global population, wheat is a grain of strategic importance^6,7^. It’s a fundamental part of the global food chain, requiring a comprehensive examination of its production’s sustainability^8,9^. In the 2022/23 marketing year, Türkiye ranked 10th in global wheat production and 9th in exports, with approximately 6.6 million hectares of land dedicated to wheat cultivation^10^. Consequently, there’s a growing need for new approaches to evaluate energy efficiency, resource management and environmental impacts in wheat production^11,12^.

There are numerous studies in the literature on energy, efficiency and costs in wheat production^13–15^. A study by Canakci et al. (2005) examined energy use during the production of wheat, cotton, corn and various vegetables in Antalya. They calculated the total energy input for wheat as 18680.8 MJ/ha, with the highest shares attributed to fertilizers (54.1%), seed (25.2%) and diesel (17.4%). The highest energy requirements were for land preparation and irrigation. The study concluded that wheat was more energy efficient than most other crops^16^. A similar study modeled energy consumption and greenhouse gas (GHG) emissions for rain fed wheat production using data from 140 farmers in Antalya. It found that chemical fertilizers accounted for the highest energy consumption and GHG emissions. The study suggested that optimizing energy consumption could lead to a 14% saving in total energy use and a 17% reduction in GHG emissions^17^. Yıldız (2016) analyzed the energy input-output and economic profitability of wheat production in the Çarşamba district of Samsun. The total energy input was determined to be 35737.13 MJ/ha, with the highest shares coming from diesel fuel (44.61%), followed by chemical fertilizers (23.54%) and irrigation water (10.58%). Due to the high energy input, the production was deemed unprofitable. The study emphasized that the intensive use of machinery, diesel and chemical fertilizers leads to environmental problems and highlighted the need to promote energy efficient practices^18^.

The effects of different sowing methods (traditional flat and raised bed planting) on the energy balance of wheat production in Türkiye’s Central Anatolia region have also been studied. Energy inputs were lower for raised bed planting (26.38 GJ/ha) than for traditional planting (26.51 GJ/ha). However, energy use efficiency was higher in traditional planting (7.15) compared to raised bed planting (5.39). Grain yield was higher with traditional planting, while thousand grain weight was greater with raised bed planting. In both methods, fertilizers contributed the most to the total energy input. The study noted that selecting the right variety is critical for the success of raised bed planting^19,20^. A comparison of energy use efficiency and economic analysis for wheat and sunflower production in the Thrace Region of Türkiye found that the total energy input for wheat was 23,231 MJ/ha, while for sunflower it was 10,139 MJ/ha. Energy use efficiency was 3.52 for wheat and 3.77 for sunflower. Diesel fuel accounted for the highest energy input in sunflower (59.98%), while fertilizers dominated in wheat (53.50%). Although both crops were profitable, with profit to cost ratios of 1.20 and 1.02 respectively, wheat production was found to be more energy and economically efficient^20^.

Diesel fuel and chemical fertilizers used in wheat production account for a large portion of energy consumption, which increases costs for farmers and causes environmental problems. To address these issues and ensure a sustainable future, renewable sources like wind and solar energy should be used in agricultural machinery and irrigation systems. Furthermore, producing biomass energy from agricultural waste is another important alternative in this field.

While most existing studies focus on energy efficiency in wheat production, evaluating the energy, exergy and environmental impacts of wheat production processes with a holistic approach is critical for achieving sustainable agriculture goals. Although energy balance studies provide a basis for assessing the efficiency and environmental impacts of production systems, exergy analysis, based on the second law of thermodynamics, stands out as a powerful tool for evaluating the true efficiency of a system’s energy and resource use^21,22^. Exergy analysis identifies the potential work loss and irreversibilities in energy conversion processes, revealing a system’s actual efficiency^23^.

Studies in the literature have also examined agricultural products using exergy based analyses^24–27^. Cumulative Exergy Consumption (CExC) is a concept that determines the efficiency of resource use by representing the total exergy consumed by all direct and indirect inputs during a product’s production process. Based on the CExC value, the Cumulative Degree of Perfection (CDP) and the Renewability Index (RI) can be calculated to determine the efficiency of the production system^54^. A high CDP indicates high exergetic efficiency^28,29^.

Taki and Yıldızhan (2018) used exergy analysis to evaluate greenhouse cucumber production in Iran. They stated that the CDP value increased when renewable energy sources were included^30^. In another study that performed an exergy based analysis of strawberry production in both greenhouses and open fields, the CDP values were calculated as 0.29 for open field and 0.18 for greenhouse production. The study stated that reducing cumulative exergy consumption is necessary to increase the cumulative degree of perfection and decrease exergy loss. This can be achieved by using renewable energy sources instead of fossil fuel based energy consumption^31^. Hesampour et al. (2022) found that the RI and CDP values for date production were 0.62 and 2.68, respectively. They noted that high carbohydrate and low water content increased the chemical exergy value in the calculations^32^.

Recent advances in digital and remote sensing technologies have improved the monitoring of wheat growth and resource use. Wang et al. (2024) developed a cross modal segmentation model using multi temporal satellite images and Digital Elevation Models (DEM) data for winter wheat mapping, demonstrating the potential of data driven tools to enhance precision and sustainability in agricultural management^33^. At the biological and modeling levels, sustainability is influenced by both plant resilience and data driven management. Wang et al. (2023) demonstrated that the heat shock protein TaHSP17.4 enhances wheat stress tolerance^34^, while Sha et al. (2025) developed the ZHPO-LightXBoost model for predicting pesticide residues, showing the role of intelligent systems in improving agricultural sustainability^35^.

The novelty of this study lies in the use of exergy based environmental indicators in conjunction with regional sustainability metrics (CDP and RI) to assess resource efficiency in wheat production, an approach rarely considered in current agricultural energy research.

This study introduces a significant innovation by evaluating the energy and exergy performance of wheat production in Türkiye and combining it with sustainability and environmental impact analyses. While most of the existing literature focuses on energy consumption and efficiency, this article adopts a holistic approach using thermodynamic metrics such as cumulative energy consumption (CEnC), CExC, cumulative carbon dioxide emission (CCO_2_E), CDP and RI. Additionally, the comparative analysis of the same crop in different geographical regions, Samsun and Tokat, provides concrete data on the critical role of regional differences in agricultural inputs, mechanization levels and environmental impacts. This approach allows for the creation of specific recommendations for tailoring agricultural policies to regional conditions.

## Materials and methods

This study uses a holistic approach to cumulative exergy analysis to identify exergy losses in wheat production processes and pinpoint potential areas for system improvement. The research provides new indicators to evaluate the sustainability of agricultural production and resource use efficiency based on the thermodynamic properties of wheat plants in different regions in Türkiye.

The quantitative input data used in this study are based on comprehensive field assessment studies on wheat production in the Tokat and Samsun regions. Studies by Cicek et al., (2011) and Yildiz, (2016) provide concrete technical data on key actions that support sustainable development, such as water and power use in agricultural activities, land management and economic profitability. The input data used in this study were obtained through field surveys and farmer interviews conducted in Samsun and Tokat provinces. For Samsun, data were collected from 54 randomly selected wheat producers, while in Tokat, data were obtained via face to face interviews with wheat farmers across multiple districts. These field based datasets reflect the average production practices in each province and are considered representative of local conditions. For Tokat, irrigated wheat production yielded the highest share of diesel (53.02 lt/ha), irrigation water (3087 m^3^/ha) and nitrogen fertilizer (52 kg/ha), along with total energy consumption (13,205.90 MJ/ha)^14^. For Samsun, the highest total energy consumption per ha was 35,737.13 MJ/ha. Here, the effective inputs are diesel (283.12 lt/ha), irrigation water (6000 m^3^/ha) and nitrogen fertilizer (103.10 kg/ha), similar to those in Tokat province^18^. In this study, inputs used in the wheat production process were analyzed from tillage to harvest. Post-harvest operations and storage stages were excluded from the analysis. Determining regional energy intensities reinforces the reliability of the input data used in our study and their suitability for regional conditions. This methodology increases the effectiveness of our results in supporting current agricultural practices.

According to agricultural data, the 10-year average wheat yield in Türkiye is approximately 287 kg/da^36^. In recent years, yields in Samsun have been 270 kg/da^37^, while in Tokat, it has been 253 kg/da^38^. The literature review indicates that energy, exergy and environmental analyses in agriculture are calculated cumulatively. Therefore, in accordance with the literature, analyses were conducted in ton rather than land area. The material inputs and their quantities used in the analysis are presented in Table 1. The inputs considered in the calculations include chemical fertilizers, water for irrigation, diesel fuel consumption and agricultural chemical. The quantitative data for these inputs were compiled from existing studies in the literature for Tokat^14^ and Samsun^18^. This data was adapted to the specific agricultural conditions in these provinces, with the aim of obtaining reliable results on the energy and environmental performance of regional wheat production.

**Table 1:** Input/output values in the production of one ton of wheat^14,18^.

Inputs/output		Unit	Quantity per ton	
			Samsun^14^	Tokat^18^
**Fertilizers**	Nitrogen (N)	kg	25.85	15.25
	Phosphorus (P_2_O_5_)	kg	12.14	16.13
**Chemicals**	Herbicides	kg	0.33	0.59
**Diesel**		MJ	59.64	17.19
**Water for irrigation**		kg	1504619.18	905278.59

In this study, the inputs for the wheat production process in the provinces of Tokat and Samsun were calculated based on the input values presented in Table 2. This included agricultural inputs such as fertilization, chemicals, diesel and irrigation water. The results provide a more detailed look at the energy intensity, resource consumption efficiency and environmental impact of wheat production.

**Table 2:** Specific energy, exergy and carbon dioxide emission of input values.

Inputs	Specific CEnC	Specific CExC	Specific CCO_2_E
**Fertilizers**			
Nitrogen (N)	78.2 MJ/kg^39^	32.7 MJ/kg^40^	0.09 kg/kg^41^
Phosphorus (P_2_O_5_)	17.5 MJ/kg^42^	7.52 MJ/kg^42^	0.15 kg/kg^41^
**Chemicals**			
Herbicides	198.8 MJ/kg^43^	32.7 MJ/kg^40^	6.3 kg/kg^44^
**Diesel**	57.5 MJ/kg^43^	53.2 MJ/kg^40^	0.94 kg/kg^44^
**Water for irrigation**	0.00102 MJ/kg^43^	0.00425 MJ/kg ^45^	0.000595 kg/kg^45^

### The importance of sustainability indicators

CEnC can be insufficient for a comprehensive environmental assessment because it overlooks non-energy raw material inputs during production. To address this limitation, CExC is used as a more comprehensive indicator that represents the total exergy value of resources consumed at every stage of a product’s production process^40,46^. Based on CExC value, CDP and RI are calculated. These metrics help determine the exergetic efficiency of a production method by assessing the rate at which non-renewable resources are used. Additionally, CCO_2_E are used to evaluate the environmental impact of agricultural production^48^.

### Thermodynamic analysis method

To analyze the thermodynamic performance of wheat production, we based our approach on mass, energy, exergy and entropy balance equations. From these equations, we calculated key sustainability indicators CEnC, CExC, CCO_2_E, CDP and RI using the formulas presented below^40,46,49,50^.

The mass balance is calculated as shown in Eq. (1):

1 $$\:{\Sigma\:}\left(m\right)_{\text{i}\text{n}}\:=\:{\Sigma\:}\left(m\right)_{\text{o}\text{u}\text{t}}$$

Where *m*_*in*_ (kg) and *m*_*out*_ (kg) represent the mass entering and leaving the system, respectively.

Energy balance calculations are performed as shown in Eq. (2):

2 $$\:{\Sigma\:}\left(mh\right)_{\text{i}\text{n}}\:-{\Sigma\:}\:\left(mh\right)_{\text{o}\text{u}\text{t}}\:=\:Q\:-\:W$$

Where h (kJ/kg), W (kJ) and Q (kJ) represent enthalpy, work and heat energy, respectively.

To calculate the entropy balance, Eq. (3) is used:

3 $$\:{\Sigma}\:\text{S}_{\text{g}\text{e}\text{n}\text{e}\text{r}\text{a}\text{t}\text{i}\text{o}\text{n}}\:={\Sigma}\:\left(ms\right)_{\text{o}\text{u}\text{t}}\:-\:\:{\Sigma}\left(ms\right)_{\text{i}\text{n}}\:-{\Sigma\:}\:\frac{{Q}_{\text{k}}}{{T}_{\text{k}}}$$

Here, S represents entropy, k represents the heat resource index and b represents the flow availability of stream. In addition, Q_k_ represents the heat energy entering the system and T_k_ represents the system temperature.

The exergy balance is calculated as shown in Eq. (4):

​

4 $$\:{\Sigma}\:\left(mb\right)_{\text{i}\text{n}}\:-\:{\Sigma}\:\left(mb\right)_{\text{o}\text{u}\text{t}}\:+{\Sigma}\:(1-\:\frac{{T}_{\text{o}}}{{T}_{\text{k}}})Q_\text{k}\:-\:W\hspace{0.17em}=\hspace{0.17em}\text{X}_{Ioss}$$

 

Where b (kJ/kg),​ T_0_ (K), ​ T_k_ (K) and X_loss_(kJ) represent exergy flow, ambient temperature, source temperature and exergy loss, respectively.

The flow exergy (b) is calculated using Eq. (5):

5 $$b\,=\,{b^{ch}}+{\text{ }}{b^{th}}$$

Here, *b*^*ch*^​ is used to calculate chemical exergy and *b*^*th*^​ is used for physical exergy. The calculation for physical exergy is performed using Eq. (6):

6 $$\:b^{\text{t}\text{h}}\:=\:R_{\text{u}}\:T_0\:{\Sigma}_\text{i}\:y_\text{i}\:\text{l}\text{n}\:\left(y_\text{i}\right)\:$$

An important measure of a system’s efficiency in the production process, the CDP value, is calculated using Eq. (7)^40^:

7 $$\:CDP=\frac{{\left(\text{m}\text{b}\right)}_{\text{p}\text{r}\text{o}\text{d}\text{u}\text{c}\text{t}}}{\sum\:{\left(m\text{C}\text{E}\text{x}\text{C}\right)}_{\text{r}\text{a}\text{w}\:\text{m}\text{a}\text{t}\text{e}\text{r}\text{i}\text{a}\text{l}\text{s}\:}+\sum\:{\left(m\text{C}\text{E}\text{x}\text{C}\right)}_{\text{f}\text{u}\text{e}\text{l}\text{s}\:}}$$

​In thermodynamic analyses, CExC is a crucial concept for evaluating the sustainability performance of systems. Expanding on this concept, Berthiaume and Bouchard (1999) defined new indicators such as net exergy consumption (CENx) and restoration work (W_r_)^46^. According to this approach, net exergy consumption can also be expressed as the total cumulative exergy loss that occurs in a process, allowing for a more comprehensive understanding of a system’s efficiency and environmental impact^46^.

8 $$\:CNEx=CExC-{Ex}_{p}$$

Where Ex_p_ represents the chemical exergy of the product. *W*_*r*_ is calculated using Eq. (9)^47^:

9 $$\:{W}_{r}={CNEx}_{p}-{CNEx}_{waste}$$

Where CNEx_p_ and CNEx_waste_ ​refer to the non-renewable energy consumption and energy for waste treatment, respectively. The RI is an indicator determined by the ratio of useful work produced to restoration work and it can be calculated using Eq. (10) to determine the renewability of a production system^47^:

10 $$\:RI=\frac{{W}_{P}-{W}_{r}}{{W}_{p}}$$

Where W_p_ represents the useful work obtained from the product.

Important scenarios for different RI value ranges are defined in Table 3.

**Table 3:** Definition of processes based on RI ranges^47^.

RI Value	Process Description
RI = 1	Fully renewable process (W_r_ = 0)
0 < RI < 1	Partially renewable process
RI = 0	Work produced is equal to restoration work
RI < 0	Restoration work is greater than the produced work

**Fig. 1:**
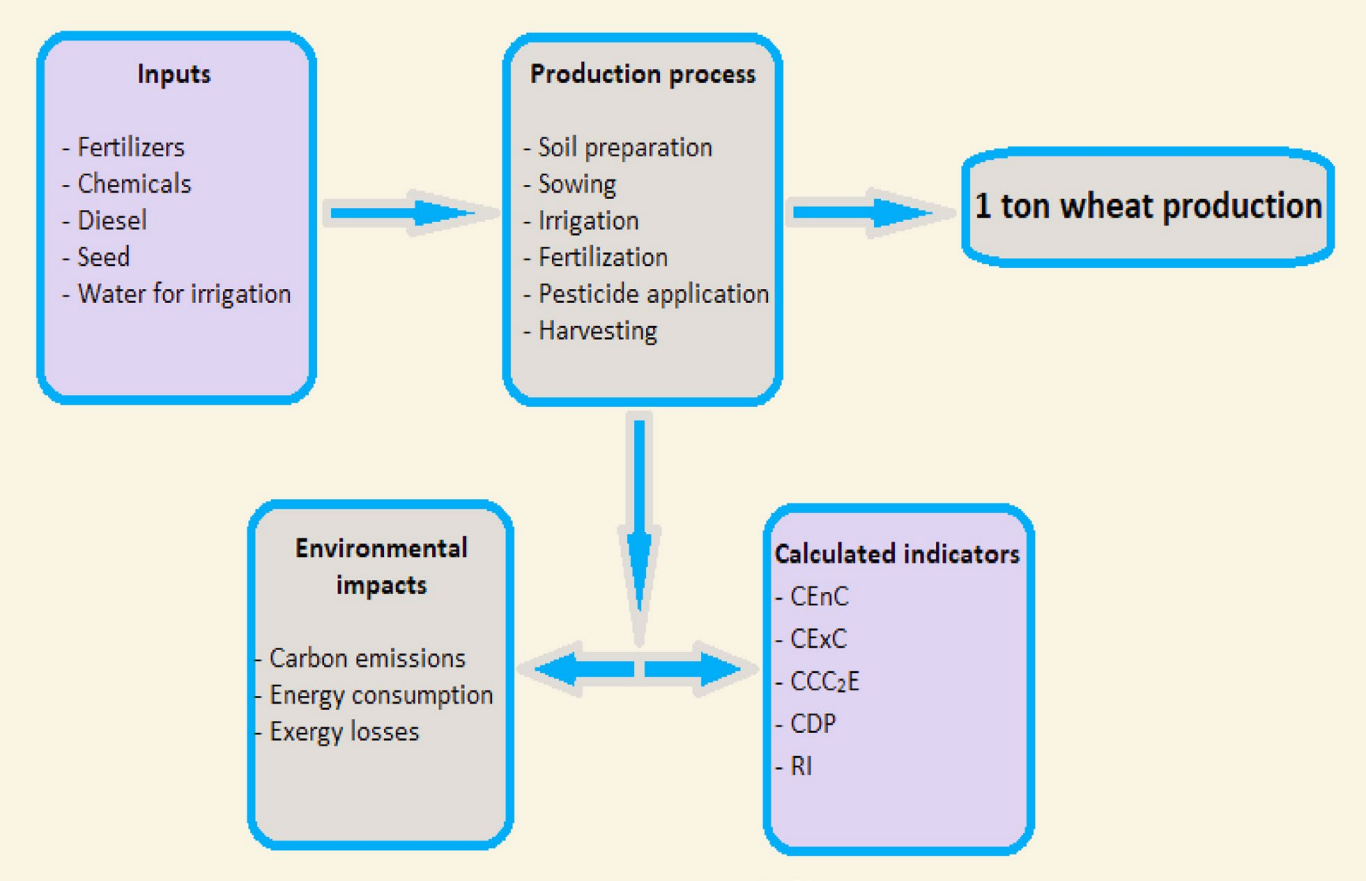
System boundary and investigation procedure for the wheat production.

In this study, the system boundaries were established for the production of 1 ton of wheat and the system’s energy, exergy and environmental performance were analyzed in detail. The boundaries shown in Fig. 1 include only the agricultural production stages, excluding post-harvest processes such as transportation, processing and storage. First, all necessary inputs for production were identified and their corresponding energy, exergy and CO₂ values were calculated. Using this data, indicators such as CEnC, CExC, CCO_2_E, CDP and RI were evaluated. This approach aims to provide a more holistic view of the efficiency of resource use and the environmental impact of wheat production.

## Results and discussion

The results of this study were calculated using the input data for 1 ton of wheat production from Table 1 and the specific energy, exergy and carbon dioxide emission values presented in Table 2. These calculations enabled a comprehensive assessment of the energy and environmental impacts at each stage of the production process.

The analysis provides a detailed breakdown of how inputs used in wheat production contribute to energy consumption, exergy losses and greenhouse gas emissions. The production process’s total cumulative energy, exergy and carbon dioxide emission values were determined. These cumulative values serve as a fundamental reference for evaluating the system’s energy and environmental efficiency and they are important indicators for future comparisons with different production methods or agricultural practices.

### Cumulative energy consumption (CEnC) analysis

**Fig. 2:**
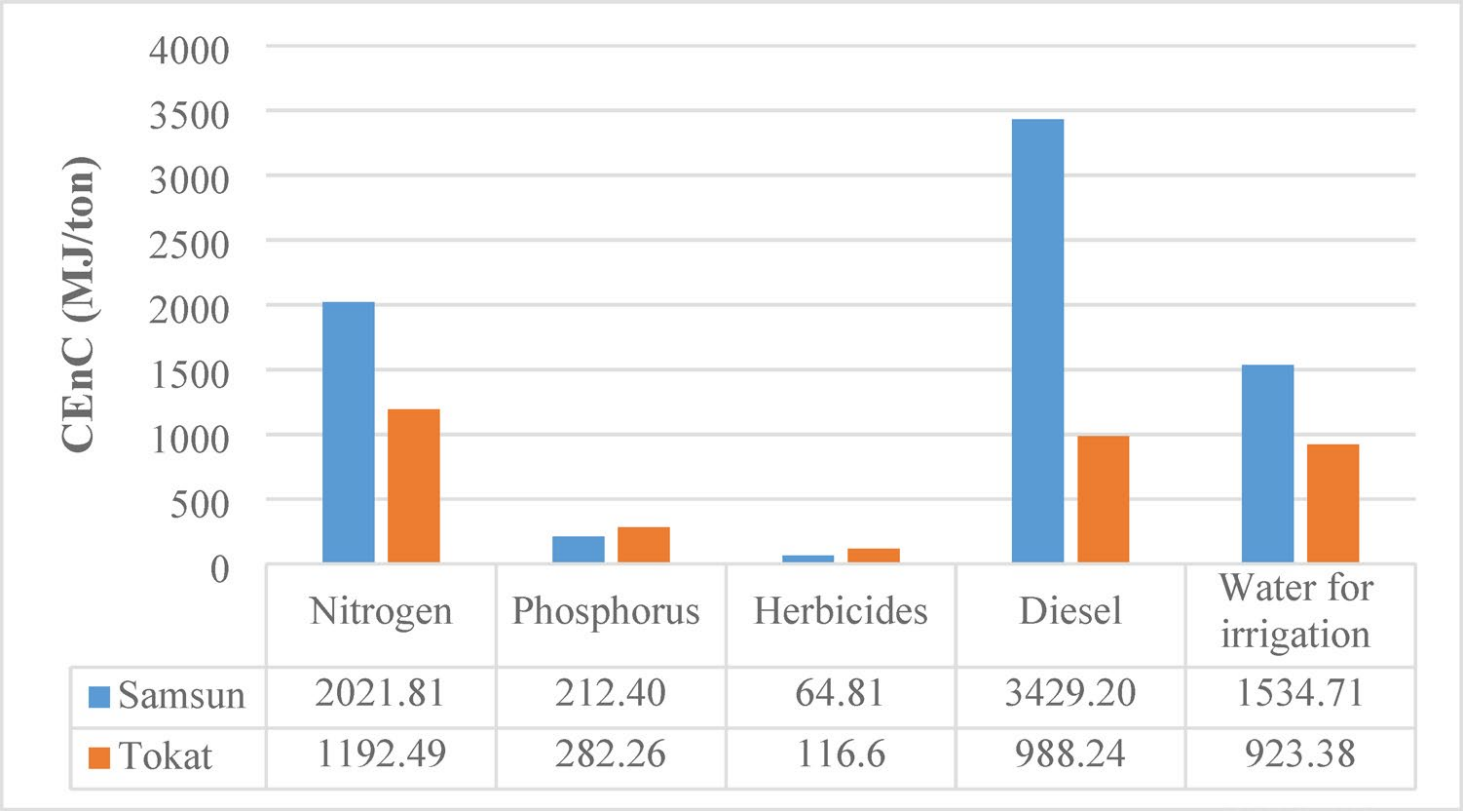
CEnC results of the wheat production process.

The CEnC (MJ/ton) for various inputs used in wheat production was analyzed comparatively for Samsun and Tokat provinces. The total CEnC is 7262.93 MJ/ton in Samsun and 3502.97 MJ/ton in Tokat, meaning that wheat production in Samsun is approximately twice as energy intensive as in Tokat. As shown in Fig. 2, the most significant consumption comes from diesel, irrigation and nitrogen fertilizer use. Diesel consumption is the highest energy input in Samsun, at 3429.20 MJ/ton, while it is only 988.24 MJ/ton in Tokat. This difference suggests that mechanization is more intensive in Samsun or that agricultural activities there consume more fuel. Diesel’s share of total energy consumption is around 47% in Samsun and just 28% in Tokat. Nitrogen fertilizer consumption is 2021.81 MJ/ton in Samsun and 1192.49 MJ/ton in Tokat. This input’s share of the total energy is about 27.8% in Samsun and 34% in Tokat. This shows that nitrogen fertilizers are the second most energy intensive input in both regions.

Irrigation water energy consumption was calculated as 1534.71 MJ/ton in Samsun and 923.38 MJ/ton in Tokat. In Samsun, irrigation’s share of total energy consumption is 21%, while it’s 26% in Tokat. This indicates that even though water use is lower in Tokat, it’s a relatively more significant energy input there compared to Samsun. Phosphorous fertilizer consumption is 212.40 MJ/ton in Samsun and 282.26 MJ/ton in Tokat. Tokat’s higher consumption value is noteworthy, though its share of total energy is only about 8% compared to Samsun’s 3%. Herbicide use is 64.81 MJ/ton in Samsun and 116.60 MJ/ton in Tokat. While Tokat’s consumption of this input is nearly double Samsun’s, its contribution to total energy consumption is very low and has a limited impact on the overall energy balance.

Many studies in the literature have examined the energy consumption of various agricultural products. The inputs responsible for the most significant energy consumption have been identified in numerous studies. Using similar analysis methods, Özilgen and Sorgüven (2011) calculated the cumulative energy consumption for olive, sunflower and soybean as 8533.2 MJ/ton, 5179.2 MJ/ton and 6072.7 MJ/ton, respectively^29^. Değerli et al. (2015) compared the energy consumption for wheat bread production in Türkiye and Germany, reporting values of 5.4 MJ/kg and 1.9 MJ/kg, respectively^23^. This shows that wheat production in Türkiye uses about 2.8 times more energy than in Germany, highlighting significant opportunities for improvement in Türkiye’s wheat production process. In a study on wet and dry wheat production in Iran, Yıldızhan and Taki (2019) calculated the cumulative energy consumption as 5044.54 MJ/ton for wet and 5520.46 MJ/ton for dry production^51^. The findings from these studies, which show higher consumption from diesel and nitrogen based inputs, are consistent with this current study’s results.

Overall, the energy intensity of wheat production in Samsun is significantly higher than in Tokat. The main reason for this difference is diesel consumption. As a result, wheat production in Samsun has a high energy intensive structure with a greater environmental impact, while Tokat presents a more energy efficient and sustainable production model. This finding indicates that regional agricultural mechanization levels, climate and soil conditions play a critical role in energy consumption. Therefore, it can be concluded that Tokat offers a more advantageous production model in terms of energy efficiency, while Samsun needs improvements, especially in its use of fuel and fertilizer. These findings suggest that Samsun should focus on reducing diesel consumption, improving fertilizer use efficiency and modernizing irrigation techniques.

### Cumulative exergy consumption (CExC) analysis

**Fig. 3:**
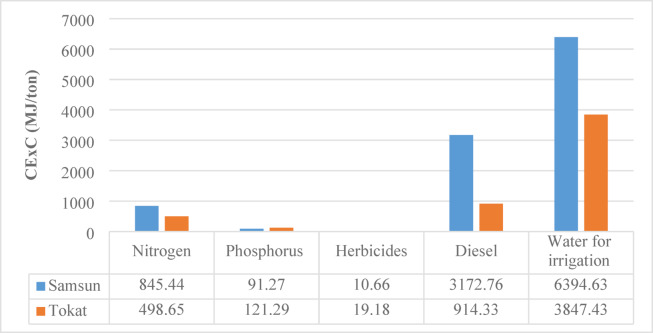
CExC results for 1 ton of wheat production.

Figure 3 provides a comparative analysis of the cumulative exergy consumption for wheat production in Samsun and Tokat provinces, broken down by input. Exergy analysis is crucial for sustainability because it considers not only the amount of energy but also its availability for use. The results show that Samsun’s total exergy consumption is much higher than Tokat’s. Samsun’s total cumulative exergy value is 10514.76 MJ/ton, while Tokat’s is 5400.88 MJ/ton. The primary reason for this difference is the high exergy consumption from irrigation water and diesel use.

Irrigation water consumption was calculated at 6394.63 MJ/ton in Samsun and 3847.43 MJ/ton in Tokat. This input was identified as the largest exergy consumer in both provinces, accounting for about 60.8% of total exergy in Samsun and 71% in Tokat. These results show that the efficiency of irrigation methods directly determines exergetic efficiency.

Diesel consumption is 3172.76 MJ/ton in Samsun and 914.33 MJ/ton in Tokat. Diesel use constitutes about 30% of total exergy consumption in Samsun but only 17% in Tokat. This suggests that agricultural mechanization is more intensive in Samsun, leading to less efficient energy use. Nitrogen fertilizer consumption is 845.44 MJ/ton in Samsun and 498.65 MJ/ton in Tokat, with a similar share of exergy consumption 13% in both regions. Phosphorous fertilizer consumption is 91.27 MJ/ton in Samsun and 121.29 MJ/ton in Tokat. Its contribution to the total exergy is less than 3%, but it is higher in Tokat than in Samsun. Herbicide use was calculated as 10.66 MJ/ton in Samsun and 19.18 MJ/ton in Tokat. The exergy load is very low, making up less than 1% of the total exergy consumption in both provinces.

In a study evaluating inputs for agricultural production, Özilgen and Sorgüven (2011) calculated exergy consumption values for olive (10762.6 MJ/ton), soybean (9051.4 MJ/ton) and sunflower (8819.1 MJ/ton)^29^. Similarly, Değerli et al. (2015) calculated the CExC value for bread wheat production as 6.7 MJ/kg in Türkiye and 2.7 MJ/kg in Germany^23^. Just like with energy consumption, studies show that exergy consumption in Türkiye is also high.

As a result, Samsun’s wheat production system demonstrates lower sustainability due to its high exergy intensity, while Tokat’s production structure is relatively more exergetically efficient. However, to further reduce the exergy load in Tokat especially from irrigation and fertilizer use it is recommended to implement modern irrigation systems and improved fertilizer management strategies.

### Cumulative carbon dioxide emission (CCO₂E) analysis

**Fig. 4:**
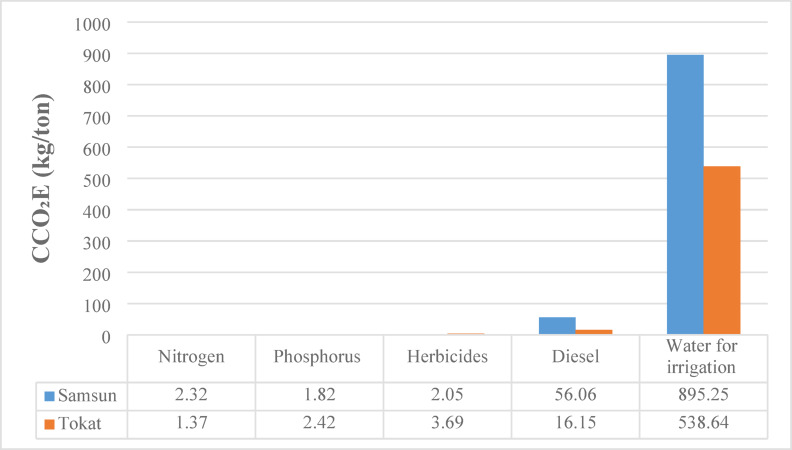
CCO_2_E results for 1 ton of wheat production.

This study calculated the cumulative carbon dioxide emission values for five different input sources used in the wheat production process in Samsun and Tokat provinces: nitrogen, phosphorus, herbicides, diesel and irrigation water. As shown in Fig. 4, the data reveals significant differences in the environmental impact of the resources used for wheat production between the two provinces. Samsun’s total cumulative carbon dioxide emission is 957.5 kg/ton, while Tokat’s is 562.27 kg/ton. When calculated based on inputs, agricultural activities in Samsun lead to approximately 58.7% more carbon dioxide emissions compared to Tokat. For both provinces, the largest source of carbon emissions is irrigation water. In Samsun, irrigation water emissions are 895.25 kg/ton, accounting for 93.5% of the total emissions. In Tokat, this value is 538.64 kg/ton, representing 95.7% of the total emissions.

Diesel use is another significant source of emissions. Emissions from diesel in Samsun are 56.06 kg/ton, which is about 3.5 times higher than the 16.15 kg/ton value in Tokat. This indicates that agricultural machinery in Samsun is either used more intensively or is less energy efficient. However, the emissions from other inputs like nitrogen, phosphorus and herbicides are relatively low in both provinces, each contributing less than 1% to the total emissions.

Hesampour et al. (2022) calculated the cumulative carbon emissions during date production as 197 kg/ton, stating that about half of these emissions came from fertilization^32^. Similarly, Yıldızhan (2017) noted that chemical fertilizers have a significant share in carbon emissions^52^. Değerli et al. (2015) also stated that carbon emissions for wheat are higher in Türkiye compared to Germany^23^.

In these studies, authors have noted that carbon footprint formation in agricultural production has different dynamics. Samsun’s emissions per ton of wheat are higher than Tokat’s, largely due to irrigation and diesel use. The data reveals that farming practices in Samsun lead to higher greenhouse gas emissions and have a greater potential for environmental improvement. In summary, to reduce the environmental impact of agriculture in both Samsun and Tokat, it’s necessary to improve irrigation methods and use more fuel efficient machinery.

### CDP and RI in wheat production

**Fig. 5:**
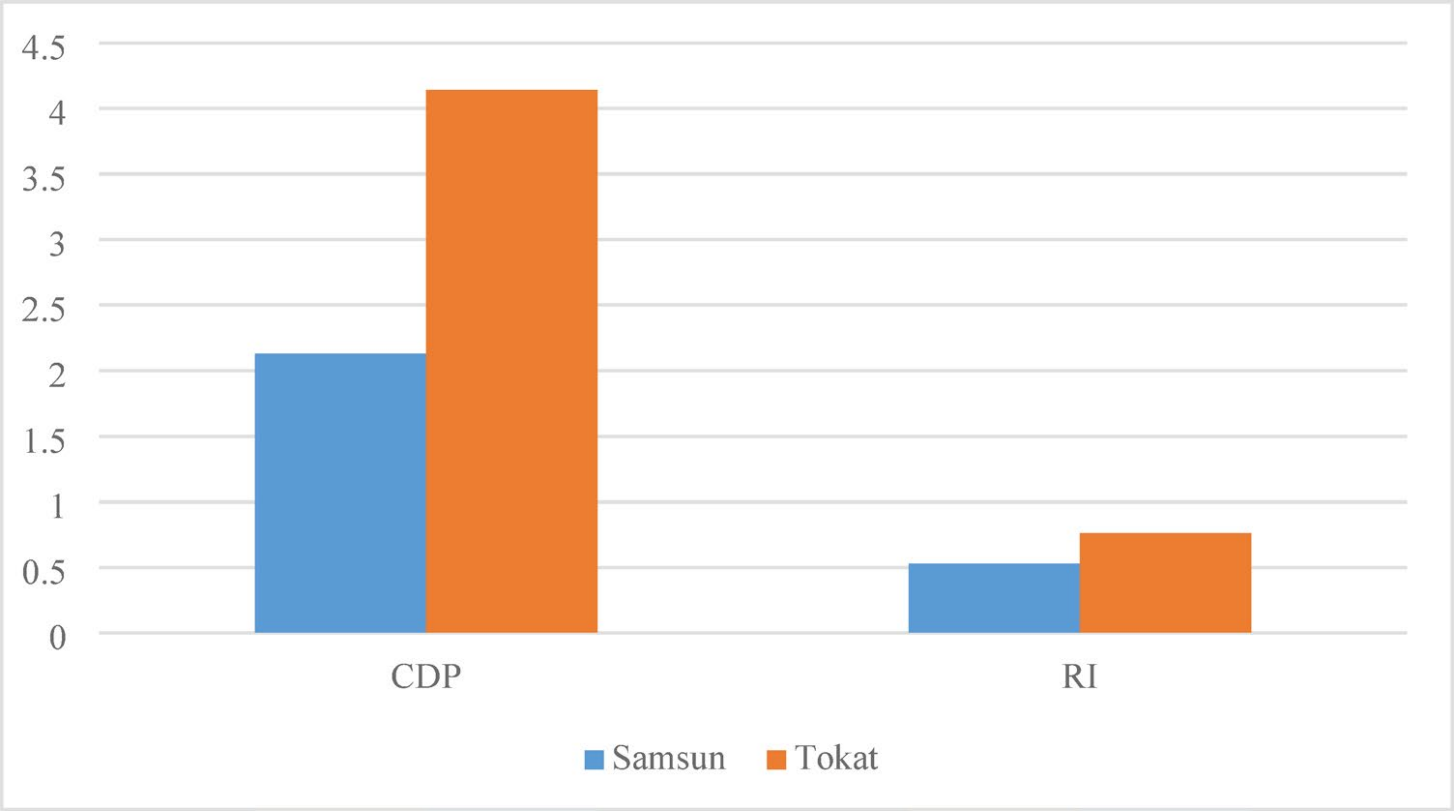
CDP and RI values ​​of provinces for wheat production.

In this study, the available exergy flow of wheat was taken as 22.36 MJ/kg from the study of Yıldızhan and Taki (2019)^51^. The sustainability performance of the wheat production processes in Samsun and Tokat was analyzed by calculating the CDP and RI values, based on the input data in Table 1. The calculated values are CDP 2.13 and RI 0.53 for Samsun and CDP 4.14 and RI 0.76 for Tokat. Figure 5 shows the calculated CDP and RI values ​​of the provinces. The CDP value provides information about the system’s exergy based efficiency, while the RI value relates to the renewability of the production process. According to the ranges provided in Table 3, the RI value for both provinces was found to be “partially renewable.” To make the process more renewable and closer to the RI = 1 value in Table 3, both provinces need to reduce their use of irrigation water and diesel.

The results show that the wheat production system in Samsun has lower exergetic efficiency and renewability compared to Tokat. This can be explained by Samsun’s high diesel consumption, intensive irrigation water use and the resulting higher energy inputs. The data indicates that Samsun’s production process has a higher environmental impact due to its more intensive resource consumption. In contrast, Tokat has lower energy and exergy consumption, which led to higher CDP and RI values. These values reveal that Tokat offers a more sustainable model for wheat production. Therefore, improving irrigation and diesel use in Samsun by adopting modern irrigation techniques and preferring more fuel efficient machinery is critical for increasing both the CDP and RI indicators.

Many authors have calculated the CDP and RI values for agricultural products in the literature. For different rice varieties, the highest CDP values were calculated as 7.96 for the Luna and 7.63 for the Barone varieties. The RI for these same rice varieties was calculated as 0.88^24^. For rapeseed, a study using an optimal scenario and new strategies calculated CDP and RI values of 3.87 and 0.92, respectively^53^. Taki and Yıldızhan (2019) calculated CDP values of 2.9 and 6.48 for wet and dry wheat production in Iran, respectively, with corresponding RI values of 0.65 and 0.84^51^. The CDP and RI values for wheat production in Türkiye are similar to those found in the literature.

This analysis not only evaluates the energy and exergy based performance of wheat production but also provides a holistic perspective on the environmental impacts of the production process. The indicators also allow for comparisons with similar agricultural products, providing a basis for examining the effects of regional differences on energy efficiency. The results from this study are expected to guide efforts to promote the use of renewable energy, optimize inputs and develop energy efficiency policies for sustainable wheat production.

A detailed input based assessment was carried out to determine how the major inputs diesel fuel, irrigation water, fertilizers and electricity affect the sustainability indicators of wheat production in Samsun and Tokat. The comparative results (Figs. 2, 3, 4 and 5) indicate that diesel fuel is the most dominant contributor to total energy consumption in Samsun, accounting for approximately 47% of CEnC. In contrast, irrigation was identified as the largest contributor to CExC in both provinces, representing 60.8% in Samsun and 71% in Tokat. This finding reflects the high energy demand of pumping systems and the exergy losses that occur during water delivery and distribution.

**Fig. 6:**
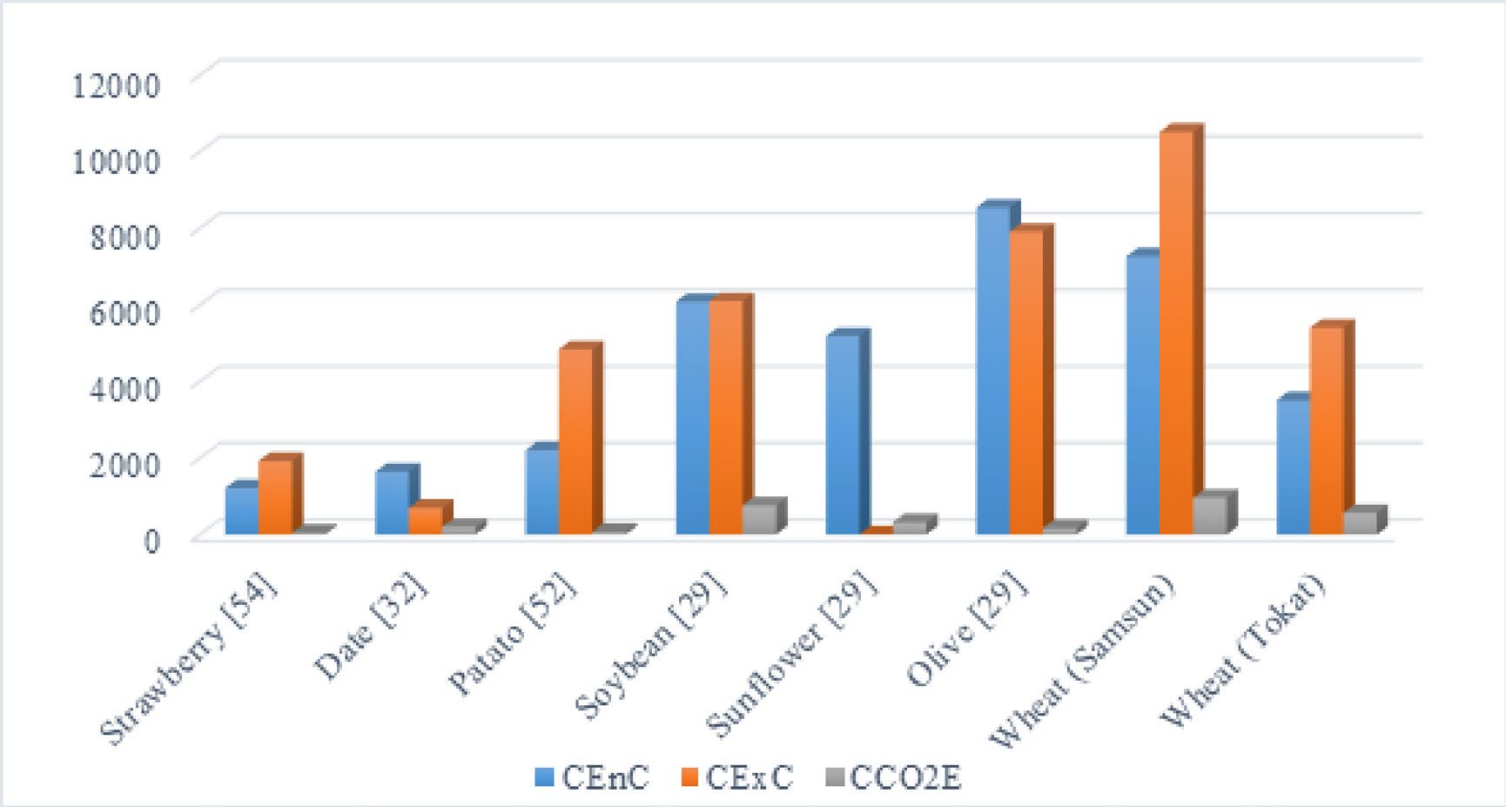
Comparison of cumulative energy, exergy consumption and carbon dioxide emissions for the present study and different products.

In terms of environmental performance, irrigation was also found to be the primary source of carbon emissions, accounting for more than 93% of CCO_2_E in both provinces. This outcome demonstrates that the sustainability of wheat production is largely determined by the energy sources and efficiency of irrigation practices. Therefore, while diesel dominates energy consumption, irrigation processes contribute most significantly to exergy destruction and environmental burdens. This input based comparison provides a clear framework for identifying which resources and processes should be prioritized to achieve higher sustainability performance in agricultural production systems. Figure 6 shows cumulative energy, exergy consumption and carbon dioxide emissions calculated for different products in comparison with the present study.

**Fig. 7:**
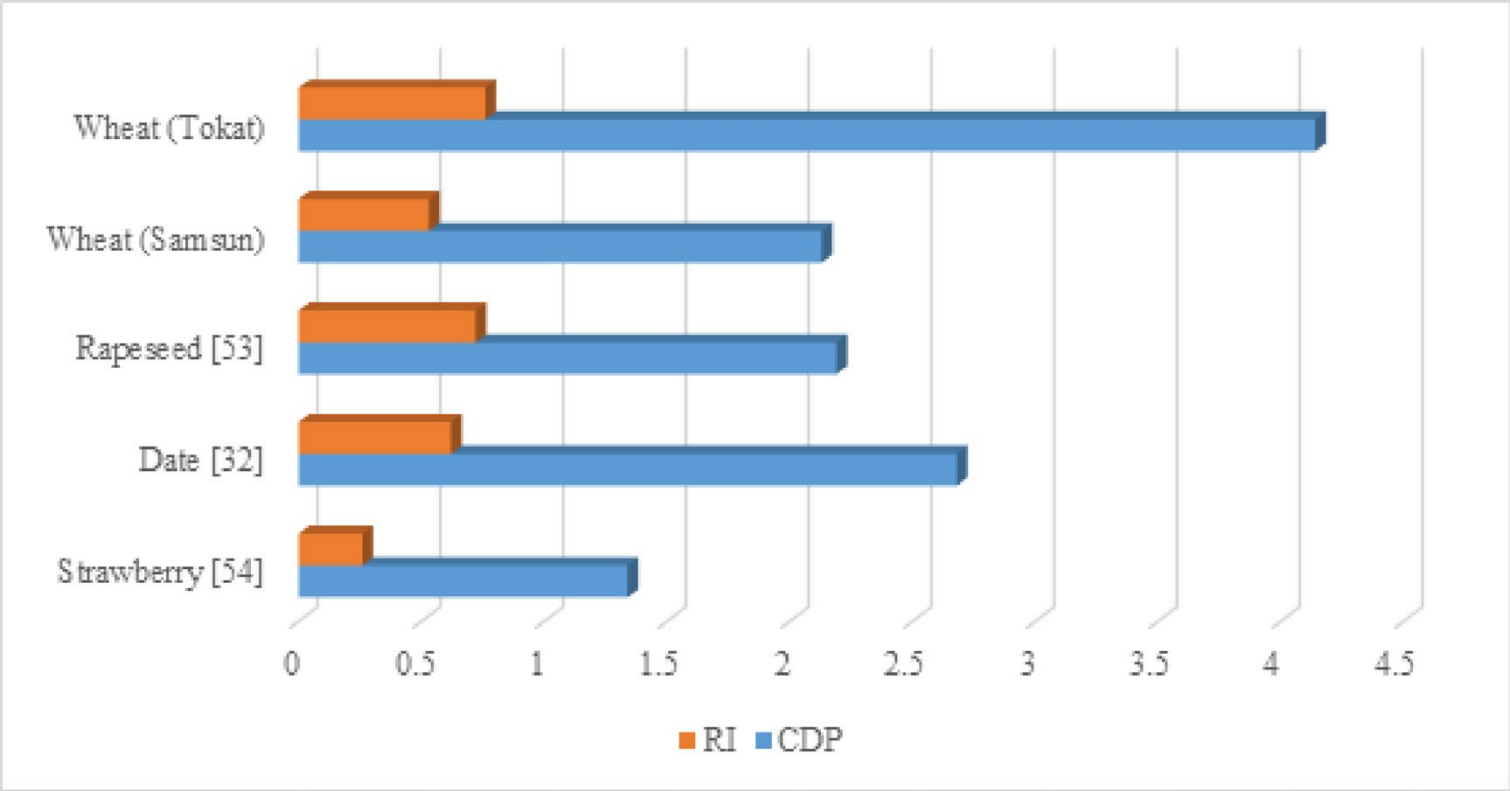
Comparison of CDP and RI values ​​of the present study and different products.

To improve the sustainability metrics (CDP and RI) of wheat production at the regional scale, tailored strategies were developed for Samsun and Tokat considering their climatic and operational characteristics. Since irrigation contributes the largest share to both energy and exergy consumption, the adoption of solar powered irrigation systems is recommended as the first step toward improvement. This measure would reduce the dependence on grid electricity and diesel for pumping, thereby lowering CO_2_ emissions and improving the renewability of the system. In Fig. 7, CDP and RI values, which are sustainability metrics for different products, are given in comparison with the present study.

Additionally, optimizing nitrogen fertilizer use and replacing a portion of mineral fertilizers with organic or bio based alternatives would help decrease fossil based exergy inputs and enhance soil quality. These improvements could have a positive impact on RI and CDP by reducing our dependency on non-renewable resources. Furthermore, the use of precision agriculture technologies, such as sensor based irrigation and fertilizer control systems, can simultaneously improve water and energy efficiency, supporting a more balanced energy, exergy and environment structure.

Overall, these region specific strategies establish a holistic framework that accounts for the ecological and socio economic diversity of Samsun and Tokat. Implementing such measures can strengthen the sustainability of wheat production by minimizing resource losses, reducing carbon emissions and promoting efficient and renewable energy utilization.

## Conclusions

This study has provided a detailed look at the energy, exergy and environmental performance of the wheat production process in Samsun and Tokat. The analysis found that wheat production in Samsun has a significantly higher energy and exergy intensity than in Tokat. This high consumption in Samsun is primarily due to the use of diesel fuel and irrigation water, which also leads to a higher environmental impact. In contrast, Tokat presents a more sustainable production model. According to the analysis, the following data was obtained:

- In Samsun, CEnC was calculated as 7262.93 MJ/ton, CExC as 10514.76 MJ/ton and CCO₂E as 957.50 kg/ton.
- In Tokat, CEnC was 3502.97 MJ/ton, CExC as 5400.88 MJ/ton and CCO₂E as 562.27 kg/ton.
- The sustainability metrics for Tokat were calculated as CDP 4.14 and RI 0.76, which were relatively higher than Samsun’s CDP 2.13 and RI 0.53. These differences point to the intensive use of irrigation and diesel.

Based on these findings, concrete steps must be taken to increase the sustainability of wheat production in Türkiye. In energy-intensive regions like Samsun, promoting modern irrigation techniques, encouraging more fuel efficient agricultural machinery and improving fertilizer management strategies are all critical for reducing total energy, exergy and carbon emissions. Additionally, using renewable energy sources for agricultural machinery and irrigation systems will help reduce dependence on fossil fuels, contributing to both economic and environmental sustainability. These results can serve as a guide for promoting sustainable practices in wheat production and supporting the transition to a green economy.

## Data Availability

The data supporting the findings of this study can be accessed from corresponding author upon reasonable request.

## Abbreviations

CCO_2_E: cumulative carbon dioxide emission, kg/ton
CDP: cumulative degree of perfection
CEnC: cumulative energy consumption, MJ/ton
CExC: cumulative exergy consumption, MJ/ton
CNEx_p_: non-renewable energy consumption
CNEx_waste_: energy for waste treatment
Ex_p_: chemical exergy
RI: renewability indicator
W_p_: useful work
W_r_: restoration work
S: entropy, kJ/(kmol K)
W: work, kJ
Q: heat, kJ
b: stream availability, kJ/kmol
h: enthalpy, kJ/kmol
m: mass, kg
T: temperature, K

## Subscripts

0: dead state
k: index of heat sources
In: inlet
out: outlet
